# Spontaneous bowel perforation due to norovirus: a case report

**DOI:** 10.1186/1757-1626-2-9101

**Published:** 2009-11-27

**Authors:** Nikhil Pawa, Andrew P Vanezis, Matthew G Tutton

**Affiliations:** 1 Department of General Surgery, Colchester University Hospital, Turner Road, Colchester, Essex, CO4 5JL, UK

## Abstract

Norovirus is the leading cause of epidemic gastroenteritis worldwide but the disease is usually self-limiting and generally only causes serious health problems in the young, elderly and immunocompromised. The authors report a case of bowel perforation in an elderly Caucasian lady with confirmed infection with Norovirus genogroup II and no other presumptive cause. To the authors' knowledge this is the first such case of bowel perforation due to Norovirus. Viral gastroenteritis should be considered in the list of differentials when no obvious cause of bowel perforation can be identified to minimise morbidity and mortality.

## Introduction

Since the developments of molecular diagnostic methods, Noroviruses have been documented as the leading cause of epidemic gastroenteritis in all age groups, causing greater than 90% of non-bacterial and approximately 50% of all epidemic gastroenteritis worldwide [1].

Colloquially known as 'gastric flu' or the 'winter vomiting bug', Norovirus is a member of the *Caliciviridae *family of viruses and is thought to be the leading cause of infectious gastroenteritis in England and Wales [2]. Initially coined 'Norwalk' virus after the small town of Norwalk in Ohio where an outbreak of acute gastroenteritis struck a primary school in the late 1960s, the virus was given its current name in 2002 by the International Committee on Taxonomy of Viruses [3].

The virus is spread by the vomitus and faecal routes and as such, there is a tendency for outbreaks to occur in enclosed spaces, namely hospitals, nursing homes, schools, offices and even cruises ships; in 2006, 679 people on the cruise ship 'Carnival Liberty' contracted the virus [4] and more recently in July 2009, 380 of the 769 passengers aboard the US cruise ship 'MS Marco Polo' on a UK round-tour contracted the virus.

The norovirus group are singled stranded RNA viruses with two main strains of the virus affecting humans. Within these two groups numerous genotypes have been detected, however it is thought that the genotype II.4 has been the culprit for a predominant number of viral gastroenteritis in the last decade [5]. The numbers of laboratory reported cases of norovirus infection shows considerable yearly fluctuation but it is thought that the vast majority of cases go unreported and as such it is difficult to determine the true scale of the disease. The majority of cases occur in the winter period. In a 12 week period in winter 2007 there were 1325 laboratory reported cases in England and Wales compared to 1845 cases in a similar time period in 2002 [6].

Norovirus gastroenteritis is usually mild and self-limiting. Studies on healthy adults demonstrate a short incubation period (24-60 hours) and infection duration (12-60 hours) with a high frequency of vomiting and diarrhoea [7]. Other symptoms attributed to the disease include headaches, low grade pyrexia, abdominal cramps and in rare cases, seizures.

## Case presentation

**Figure 1:**
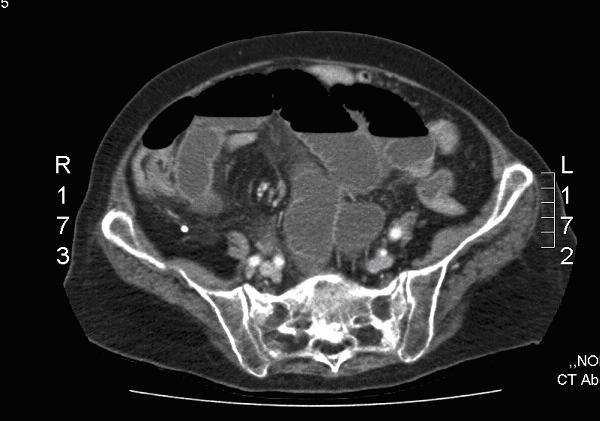
**Abdominal CT scan revealing small bowel obstruction together with mural thickening of the distal ileum**.

An 83 year old retired Caucasian lady was admitted to a UK hospital with a recent history of back pain without any trauma. She had no significant past medical history of note and her only regular medication included a statin for cholesterol. She was a non-smoker and drunk only minimal amounts of alcohol. X-rays on admission demonstrated severe osteoporosis with multiple collapsed lumbar vertebrae. Whilst awaiting a brace and social support she developed diarrhoea and vomiting. An initial stool sample was negative for clostridium difficile serotoxin. Over the next few days her clinical condition deteriorated and by day 6 her C-reactive protein was 357. She developed abdominal distension, pain and a plain x-ray showed centralised dilated small bowel loops. A subsequent CT scan of her abdomen revealed small bowel obstruction together with mural thickening of the distal ileum (see Figure 1).

Following CT the patient developed signs of localised peritonitis and underwent a laparotomy. At surgery necrosis and ischaemia was seen in a large portion of mid-ileum together with large quantities of pus and a localised perforation. A small bowel resection of 60 cm was performed. Histology revealed an acute gangrenous ileitis. Blood, urine and stool cultures were all negative but reverse transcription polymerase chain reaction (RT-PCR) of stool cultures revealed Norovirus genogroup II. Immunoassay of the stool was negative for rotavirus and adenovirus. No specific testing for astrovirus was performed. Immunological testing of the histological specimens was not performed.

The patient had no prior history of vascular disease to suggest the cause of the ileitis was ischaemic enterocolitis. Furthermore despite the patient's back pain she had been mobilising around her bed in the hospital therefore it is unlikely that the cause of the ileitis was paralytic ileus secondary to immobility. The patient received only as required opiate analgesia and this was co-administered with appropriate laxative cover to avoid constipation. A diagnosis of acute gangrenous ileitis secondary to Norovirus was therefore made. It was postulated that the virus had triggered a localised inflammatory response leading to necrosis which had continued despite clearing of the virus by host immune responses. The virus was thought to be a nosocomial infection as there was no recent history of foreign travel or contact with individuals demonstrating the symptoms of gastro-enteric disease prior to admission. However antibody testing for IgG and IgM was not performed therefore the length of infection with the virus was unclear.

Following surgery the patient developed a pelvic collection requiring CT guided drainage which matured into an enterocutaneous fistula. The fistula was treated conservatively and sealed spontaneously at 10 weeks at which point she was discharged.

## Discussion

To the best of our knowledge this is the first reported case of a small bowel perforation secondary to Norovirus infection. Even so it must be noted that many infected individuals remain asymptomatic. This is thought to be due to acquired immunity as well as the body's innate immunity. However most individuals' acquired immunity does not last through to the next season of the disease where they are again at risk of infection, thus explaining the high rates of infection in all ages [8]. Studies have now shown asymptomatic individuals to have mean viral loads similar to those of symptomatic individuals, possibly accounting for the increased number of infections and the predominance of an asymptomatic transmission route [9]. This is further supported by excretion studies performed at an aged-care facility, revealing viral shedding continuing for an average of 28.7 days [10]. Despite this the mortality rates in healthy adults is low, with most deaths occurring in the elderly, the young and the immunocompromised.

## Conclusion

The inclusion of viral gastroenteritis in the differential diagnosis of patients presenting with an acute abdomen is paramount. Potential surgical complications, particularly in high risk groups, must always be considered early to minimise morbidity and mortality.

## Consent

Written informed consent was obtained from the patient for publication of this case report and accompanying images. A copy of the written consent is available for review by the Editor-in-Chief of this journal.

## Competing interests

The authors declare that they have no competing interests.

## Authors' contributions

MT was the chief clinician looking after the patient. NP contributed to writing the case presentation and AV contributed to writing the literature review and discussion. All authors read and approved the final manuscript.

## References

[B1] Patel MM Widdowson MA Glass RI Systematic literature review of role of Noroviruses in sporadic gastroenteritis Emerg Infect Dis 2008 14 1224 31 10.3201/eid1405.071426 18680645 PMC2600393

[B2] Health Protection Agency FAQs http://www.hpa.org.uk

[B3] Dancer SJ Norovirus: an established viral plague J R Coll Physicians Edin 2008 38 314 6

[B4] Investigation update on the Carnival Liberty Centres for disease control and prevention http://www.cdc.gov

[B5] Lopman B Zambon M Brown DW The evolution of Norovirus, the 'Gastric flu' PLoS Med 2008 5 e42 doi:10.1371/journal.pmed.0050042 10.1371/journal.pmed.0050042 18271623 PMC2235896

[B6] Health Protection Report- Norovirus Update 2007 http://www.hpa.org.uk

[B7] Kaplan JE Feldman R Campbell DS The frequency of a Norwalk-like pattern of illness in outbreaks of acute gastroenteritis Am J Public Health 1982 72 1329 32 10.2105/AJPH.72.12.1329 6291414 PMC1650540

[B8] Lindesmith L Moe C Marionneau S Human susceptibility and resistance to Norwalk virus infection Nat Med 2003 9 548 53 10.1038/nm860 12692541

[B9] Ozawa K Oka T Takeda N Norovirus Infections in Symptomatic and Asymptomatic Food Handlers in Japan J Clin Microbiol 2007 45 3996 4005 10.1128/JCM.01516-07 17928420 PMC2168587

[B10] Tu ET Bull RA Kim MJ Norovirus Excretion in an Aged-Care Setting J Clin Microbiol 2008 46 2119 21 10.1128/JCM.02198-07 18417655 PMC2446857

